# Meta-Analysis of the Effects of Predation on Animal Prey Abundance: Evidence from UK Vertebrates

**DOI:** 10.1371/journal.pone.0002400

**Published:** 2008-06-11

**Authors:** Alison R. Holt, Zoe G. Davies, Claire Tyler, Samantha Staddon

**Affiliations:** 1 Catchment Science Centre, University of Sheffield, Sheffield, United Kingdom; 2 Biodiversity and Macroecology Group, Department of Animal and Plant Sciences, University of Sheffield, Sheffield, United Kingdom; 3 Centre for Evidence-Based Conservation, School of the Environment and Natural Resources, Bangor University, Bangor, United Kingdom; 4 Institute of Geography, University of Edinburgh, Edinburgh, United Kingdom; Monterey Bay Aquarium Research Institute, United States of America

## Abstract

**Background:**

Controlling vertebrate predators is one of the most widespread forms of wildlife management and it continues to cause conflict between stakeholders worldwide. It is important for managers and policy-makers to make decisions on this issue that are based on the best available scientific evidence. Therefore, it is first important to understand if there is indeed an impact of vertebrate predators on prey, and then to quantify this impact.

**Methodology/Principal Findings:**

Using the UK as a case study, we use a meta-analytical approach to review the available evidence to assess the effect of vertebrate predation on animal prey abundance. We find a significant effect of predators on prey abundance across our studies. On average, there is a 1.6 fold increase in prey abundance in the absence of predation. However, we show significant heterogeneity in effect sizes, and discuss how the method of predator control, whether the predator is native or non-native, and aspects of study design, may be potential causes.

**Conclusions/Significance:**

Our results allow some cautious policy recommendations to be made regarding the management of predator and prey populations. Meta-analysis is an important tool for understanding general patterns in the effect of predators on prey abundance across studies. Such an approach is especially valuable where management decisions need to be made in the absence of site-specific information.

## Introduction

Controlling vertebrate predators is one of the oldest and most widespread forms of wildlife management [1]. However, its use is contentious due to differences in the way that both prey and predator are valued by stakeholders, especially when the prey is of economic importance and the predator is threatened or endangered [2]. In many instances, predator control has caused conflict between interest groups, and in some has formed the basis of a long-term dispute (e.g. Hen harriers (*Circus cyaneus*) [3]; Coyotes (*Canis latrans*), [1]). Therefore, the consequences of vertebrate predation on animal prey populations are important management and policy issues.

Globally, predator control is most commonly used to protect livestock and to maximise the harvesting of game [4]. Predator-livestock conflicts arise where predator control is used to minimise economic losses. An example of such antagonism is the predation of sheep, goats and cattle by coyotes in the Western USA [1], [5]. Control as a result of perceived economic losses from depredation of game species has also caused conflict throughout Europe and in North America [4]. Examples include the effect of a number of raptor species on grey partridge (*Perdix perdix*) and pheasants [6]. Predator control is also increasingly employed to protect prey species of conservation concern from both native and non-native predators [7]. Despite the conservation goal, this practice can also cause conflict, particularly with animal rights groups [8].

Predator control is based on the assumption that a decrease in predators will increase prey, or at least reduce the overall losses of prey [9]. There has been much debate in the ecological literature over whether predation does indeed limit prey populations [10]. Early work suggested that vertebrate predators do not have large impacts on their prey [11]–[12]. This reflects the compensatory mortality hypothesis where predators consume the proportion of the prey population that would have suffered natural mortality in the absence of predation. Contrary to this, recent studies have shown that vertebrate predators can limit and sometimes even regulate their prey populations [13]–[19]. This is the additive mortality hypothesis based on predation causing mortality above the level of natural prey mortality. To successfully manage predator and prey populations, in order to reduce conflicts between stakeholders, it is first of all important to know if there is indeed an impact of vertebrate predators on prey, and then to quantify this impact [4]. We take the first step in this evaluation, using the UK as a case study, by tackling the basic assumption of predator control and posing the fundamental question, do vertebrate predators have an impact on their prey? In order to address this question a quantitative review of the literature is necessary. The UK is an ideal example of where predator control is employed for a variety of reasons, and it has a long history. Indeed, it has been used for over 200 years [20] and as a result there exists a relatively extensive scientific literature on which to draw.

Here, we critically appraise the available literature to quantify the effect that vertebrate predators in the UK have on the abundance of their animal prey. We explore the data to understand if there are important biological characteristics of the predator and prey species, or any aspects of the data used that may cause variation in this effect. In doing so, we assess the extent to which this quantitative approach is useful in informing management and policy decisions.

## Results

**Figure 1:**
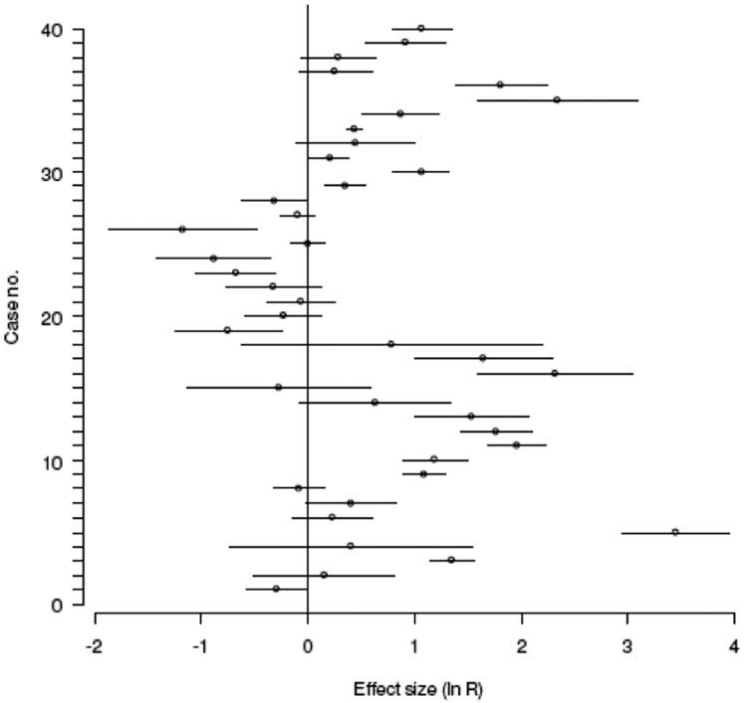
Plot of effect sizes (ln R)±SE for each of the forty cases in the meta-data set. Overall mean effect size 0.47, df = 39, 95% CI = 0.39–0.55 (fixed effects model).

There was a significant positive mean effect size across the meta-data set (Figure 1): on average, vertebrate predator removal or absence caused a 1.6 fold increase in the abundance of prey species. Seventeen of the 27 cases with positive effect sizes were significant. The largest significant response ratio (ln R+s.d. = 3.46±0.5) of predator absence concerned the effect of American mink (*Mustela vison*) predation on the fledging of common terns (*Sterna hirundo*) in western Scotland (data from Craik 1998). The number of chicks fledged in mink-free areas was 31.7 times higher than areas with mink. Of the 12 cases that showed negative effect sizes, four were significant and one case showed a response ratio of zero.

**Figure 2:**
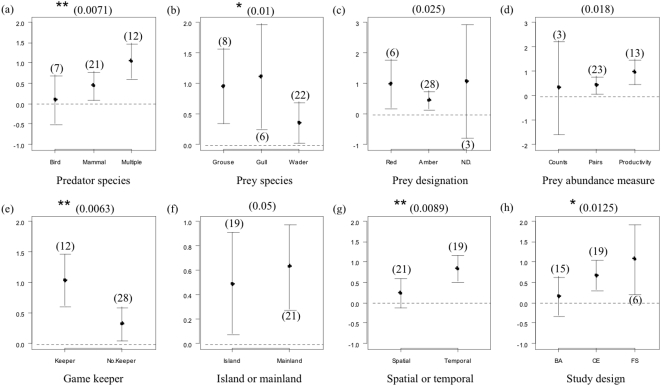
Mean effect size (ln R), confidence intervals with sample size above in parenthesis, for each group within each of the factors thought to be possible causes of heterogeneity in the meta-data set. If 95% confidence intervals are above zero (dotted line) this indicates a significant increase in prey abundance with predator control, if confidence intervals cross the line then there is no significant effect. Non-adjusted significance levels are above the graphs (* p<0.05, ** p<0.01) with Bonferroni adjusted significance levels quoted in parenthesis (the original p value would have to be below this critical value in order to be significant).

Heterogeneity in effect sizes between cases was significant (Q_T_ = 238.95 df = 39 p<0.00001), with several of the factors (Table 1) appearing to influence it. Considering predator taxon, there was a significant positive effect size for cases involving mammal predators or multiple predators, reflecting an increase in prey abundance with predator removal, (*Mammal* ln R = 0.44, df = 20, CI = 0.09–0.79; *multiple* ln R = 1.04, df = 11, CI = 0.61–1.48; Figure 2a). However, there was no significant effect of bird predation on prey abundance (ln R = 0.10, df = 6, CI = −0.50–0.70) and this led to a highly significant heterogeneity in effect sizes between groups (Q_M_ = 10.08, df = 2, p = 0.007). Prey taxon did explain some significant heterogeneity in effect size (Q_M_ = 6.49, df = 2, p = 0.04), with a significant increase in prey abundance with predator control across all groups of prey (*Grouse* ln R = 0.96, df = 7, CI = 0.35–1.57; *Gulls* ln R = 1.12, df = 5, CI = 0.26–1.97; *Waders* ln R = 0.36, df = 21, CI = 0.03–0.70; Figure 2b). Prey population status did not explain significant heterogeneity in effect size (Q_M_ = 3.71, df = 2, p = 0.15), although significant mean effect sizes were observed for species with red and amber listing (*Red* ln R = 0.99, df = 5, CI = 0.19–1.78; *Amber* ln R = 0.46, df = 27, CI = 0.16–0.76) but not for species with no designation (ln R = 1.07, df = 2, CI = −0.79–2.93; Figure 2c).

**Table 1:** Studies from which 40 cases were extracted for meta-analysis, showing predator species, prey species and a range of coded † information used in the sub-group analyses.

Study	Predator	Prey (case number)	Prey family	Prey designation	Prey abundance measure	Study with game keeper	Island or mainland study	Spatial/temporal study	Study design
(a) [39]	Multiple	(1) Black grouse	Gr	R	*	GK	M	S	CE
(b) [40]	American mink	(2) Arctic and common terns	Gu	*	Pr	NGK	Is	S	BA
									
(c) [41] *(* [*34*], [*42*] *)*	American mink	(3) Common gulls	Gu	A	Pr	NGK	Is	S	FS
		(4) Black-headed gulls	Gu	A	Pr	NGK	Is		
		(5) Common terns	Gu	A	Pr				
		(6) Herring gulls	Gu	ND	Pr				
(d) [43]	American mink	(7) Coots	*	ND	Pr	NGK	M	S	BA
		(8) Moorhens	*	ND	Pr		M		
(e) [44]	Multiple	(9) Curlew	W	A	Pa	GK	M	T	CE
**mid-term results**		(10) Golden plover	W	A	Pa		M		
		(11) Lapwing	W	A	Pa				
		(12) Red grouse	Gr	A	Pa				
(f) [45]	Hedgehog	(13) Dunlin	W	A	Pa	NGK	Is	S	BA
		(14) Lapwing	W	A	Pa		Is		
		(15) Oystercatcher	W	A	Pa				
		(16) Redshank	W	A	Pa				
		(17) Ringed plover	W	A	Pa				
		(18) Snipe	W	A	Pa				
(g ) [46]	Hedgehog	(19) Dunlin	W	A	Pa	NGK	Is	S	BA
		(20) Lapwing	W	A	Pa		Is		
		(21) Oystercatcher	W	A	Pa				
		(22) Redshank	W	A	Pa				
		(23) Ringed plover	W	A	Pa				
		(24) Snipe	W	A	Pa				
(h) [24]	Crows and gulls	(25) Curlew	W	A	Pa	NGK	M	T	CE
		(26) Golden plover	W	A	Pa		M		
		(27) Lapwing	W	A	Pa				
		(28) Oystercatcher	W	A	Pa				
		(29) Redshank	W	A	Pa				
		(30) Snipe	W	A	Pa				
(i) [47]	Hen harriers	(31) Red grouse	Gr	A	Pr	NGK	M	S	CE
(j) [48]	American mink	(32) Arctic terns	Gu	A	Pa	NGK	Is	S	FS
(k) [49]	American mink	(33) Lapwing	W	A	Co	NGK	Is	S	FS
**mid-term results**									
(l) [50]	Multiple	(34) Black grouse	Gr	R	Pr	GK	M	T	CE
		(35) Capercaille	Gr	R	Pr		M		
(m) [51] *(* [*52*] *)*	Multiple	(36) Capercaille	Gr	R	Pr	GK	M	S	CE
(n) [53] *(* [*54*] *)*	Multiple	(37) brown hares site 1	*	*	Co	GK	M	T	CE
		(38) brown hares site 2	*	*	Co		M		
(o) [55] *(* [*53*] *)*	Multiple	(39) Grey partridge site 1	Gr	R	Pr	GK	M	T	CE
		(40)Grey partridge site 2	Gr	R	Pr		M		

Citations in italics are studies that hold data that is non-independent to those from which the data in the analyses was extracted.

Multiple refers to the removal of corvid, mustelid, fox, and other mammal and bird predators. ^†^Codes: Gr/Gu/W–prey family Grouse/Gulls/Waders; R/A/ND–prey designation - red/amber/no designation (following the red and amber lists of Gregory et al. (2002)); Co/Pa/Pr–prey abundance measure–counts/breeding pairs/productivity; GK/NGK–game keeper/no game keeper sites; Is/M–island/mainland; S/T - spatial/temporal; BA/CE/FS–study design - before and after predator invasion/controlled experiment/field study with removal. The ^*^ denotes cases that could not be included in any of the groups of the categorical analysis.

Prey species abundance measure had little impact on overall heterogeneity in effect size (Q_M_ = 3.81, df = 2, p = 0.15), although it appears that the effects of predators may be more pronounced when prey abundance is measured as pairs or productivity (*Pairs* ln R = 0.43, df = 22, CI = 0.07–0.80; *Productivity* ln R = 0.97, df = 12 CI = 0.46–1.48) than as individuals (ln R = 0.33, df = 2, CI = −1.57–2.23; Figure 2d).

Predator control method has a highly significant influence on the effect of predator removal (Q_M_ = 9.41, df = 1, p = 0.002). Both keepered and non-keepered predator control resulted in a significant increase in prey abundance, with a higher increase in keepered cases ( ln R = 1.04, df = 11, CI = 0.62–1.46) than in non-keepered cases (ln R = 0.33, df = 27, CI = 0.05–0.60; Figure 2e). Both mainland and island studies showed significant mean effect sizes (*Mainland* ln R = 0.63, df = 20, CI = 0.28–0.97; *Island* ln R = 0.49, df = 18, CI = 0.08–0.91; Figure 2f), with no significant heterogeneity between groups (Q_M_ = 0.27, df = 1, p = 0.60).

Study design explained significant heterogeneity across cases, temporal studies with significantly higher effect sizes (ln R = 0.84, df = 18, CI = 0.52–1.17) than spatial studies (ln R = 0.24, df = 20, CI = −0.12–0.60; Q_M_ = 6.85, df = 1, p = 0.009; Figure 2g). Cases comparing before and after predator arrival showed no significant increase in prey abundance in the absence of predators (ln R = 0.17, df = 14, CI = −0.32–0.65; Figure 2h), whereas significant effects on prey abundance were detected for both controlled experiments and field studies removing a single predator (CE ln R = 0.68, df = 18, CI = 0.31–1.04; FS ln R = 1.07, df = 5, CI = 0.21–1.92). Overall there was a significant difference between these groups (Q_M_ = 5.83, df = 2, p = 0.05).

Analysis of the funnel plot suggested no bias in the reporting of results (see Figure S1 in supporting information). The fail-safe numbers [21] showed that 1685 non-significant studies would be needed to overturn the significant results, indicating robust meta-analyses results.

## Discussion

Our meta-analysis has shown that, on average, when vertebrate predators are removed or are absent from a system, prey abundance increases significantly. These findings are supported by the conclusions of eighty percent of the experimental studies reviewed that did not meet the criteria for meta-analysis (Table in Appendix S1). They also complement results of taxon specific reviews using worldwide data [13]–[14], [22], which have found that predators do indeed limit their prey. However, there was significant heterogeneity in the effect of predation on prey across the studies, and our sub-group analysis indicated a number of factors that might explain some of the variation. We consider these separately, although they are not mutually exclusive.

First, the effect of predation may differ depending on whether the predator is mammal or avian, the latter showing a lower, non-significant effect size. A possible explanation here is that native predators, which in this study were all avian, may have less impact on their prey than non-native predators, which were all mammal species. Prey populations may suffer compensatory mortality when predated by native predators but, conversely, mortality may be additive as a result of non-native predators. Indeed, a recent meta-analysis [23] illustrated that non-native predators can show more intense suppression of prey populations than native ones, perhaps due to prey in communities with new alien predators being predator-naïve.

However, our result may be due to predator compensation, where predation by one species is compensated for by another in the same guild. Six of the seven avian predator cases were from [24] where uncontrolled fox (*Vulpes vulpes*) predation may have replaced predation by birds. Other research has shown that when avian predators are removed their predation is often compensated for by mammal species [10], [25]–[26]. We can then assume that controlling a combination of predators (thus removing the opportunity for compensation), will have a significantly higher impact on prey than controlling either avian or mammal predators separately. Our results show that predator control by game keeper was over 3 times more effective than in cases where single predators were controlled. Not surprisingly trained game keepers are likely to perform more effective predator control which in turn may reduce the likelihood of compensatory predation.

A limitation of the results is the lack of independence between some of the factors analysed. This is especially apparent when attempting to explain the significant lower effect size of wader prey species compared to those from the grouse and gull groups. This difference may be attributed to the fact that all cases in the grouse group were from studies that used game keepers, and those from the gull group from studies with non-native predators.

Prey designation and prey abundance measure did not explain significant heterogeneity in effect size but they showed some interesting trends. It has been suggested that the impact of predator control on declining prey species may not be sufficient to reverse the trend, and prey that are increasing in abundance will continue to do so [7]. Our results run counter to this observation as the majority of species in the analyses are either red or amber listed and show positive average effect sizes. The red listed species were all from controlled experiments with a game keeper, which perhaps is a reason for these high effect sizes. As in [14], [27] we found that predators had a larger impact on productivity than on breeding population, and from a conservation perspective it is breeding population that is of importance. However, it is possible that any increase in breeding population may be masked by yearly shifts in breeding sites, and/or the action of density dependence during the breeding season [14], [28]. It, therefore, remains questionable whether the increase in abundance from predator control truly benefits prey of conservation concern.

Particular aspects of study design were responsible for some of the heterogeneity in predator impact. Temporal studies showed a significantly higher prey abundance in the absence of predators than spatial studies, perhaps because the temporal studies were on average longer than the spatial ones (temporal - 5 years, spatial - 1.67 years), giving time to capture prey response. In addition, the temporal studies were all controlled experiments with game keepers. Given the effectiveness of the controlled experiments with game keepers, we would have expected controlled experimental design to show the highest effect sizes. However, uncontrolled field studies had significantly higher effect sizes, probably due to all cases in this category originating from studies to understand the effect of a non-native predator on prey.

### Implications for management and policy

The conclusions of this review only allow us to make some cautious recommendations, due to the limited data and the problem of non-independence when exploring possible causes of heterogeneity in effect size. Although not conclusive, our analyses provide some support to the assertion that non-native predators have a greater impact on prey than native ones. Effective targeted control of predators here may be the only management option. It also suggests that predator control removing a combination of predator species through use of a gamekeeper is most effective, reducing the chances of predator compensation.

However, the results must be considered in the context of the meta-dataset. A significant proportion of the literature was not a controlled experiment or field comparison, and could not be included in the meta-analysis. In most systems it is impossible to conduct field studies or experiments at biologically meaningful scales, and it is not always considered ethical to remove predators. Consequently, the meta-analysis was limited to cases from game management and prey species conservation studies. None of the studies in the meta-data set examined the impact of predators on fish or livestock. In fact, there was a dearth of studies on the effect of abundant native predators such as foxes and large raptors, which may surprise many ecologists. However, in order for managers and decision-makers to benefit from this quantitative approach, and to help disentangle the influence of the factors we analysed here, data from well-designed long-term experiments on the impacts of a range of species of vertebrate predators on different prey groups is what is required.

### Conclusions

We have shown here that the synthesis of the scientific evidence has allowed the impact of vertebrate predator removal or absence on animal prey abundance in the UK to be quantified. Therefore, it has been useful in making some general, if cautious, management and policy recommendations. Understanding general patterns in the effect of predators on prey abundance is especially valuable where management decisions need to be made in the absence of site-specific information.

## Materials and Methods

### Systematic review

To be comprehensive, and to reduce reviewer bias, our literature search followed a strict protocol (e.g. [29], see Appendix S2 in supporting information). Relevant published and unpublished studies were identified from searching electronic databases, meta-search engines, library hand searches, reference list checks of earlier reviews and by asking relevant experts and practitioners. Seventy search terms were used from generic to species specific interactions (including marine mammals). Searching was completed in February 2006. Studies were reviewed if they were UK-based, controlled vertebrate predator removal experiments, where animal prey abundance from predator removal areas (experimental) were compared to those with predators present (control), and prey abundance had been measured using either density, individual counts, pair counts, or fledging success. Field studies which compared sites or years where a predator was present with those where it was absent were also considered appropriate. Studies that did not meet these criteria were omitted from the review.

### Meta-analysis

To prepare the accepted studies for quantitative analysis, a meta-data set was assembled, extracting the stated mean prey abundance measure for experimental and control sites or years. Sample sizes (*n*) which were either the number of years or sites for both experimental and control treatments, and the standard deviations, standard error or variance of the means for the control and experimental aspects of each study were also extracted. These data were taken from tables and figures of each study and calculated where raw data were available. Data were averaged over years or replicates where necessary and a study that included more than one species, and was deemed independent, was included separately in the analysis (referred to as cases from here on).

From 27,133 search hits 364 studies were submitted to the review. After viewing the title and abstract of each study, 97 studies were reviewed at full text, with an additional 28 studies from library searching and expert recommendations. Of these 15 contained the necessary data (mean, n, s.d.) for analysis. Nine were from peer-reviewed publications, and six from the grey literature. Sixteen studies which narrowly missed inclusion are listed in the Table in Appendix S1. In total, 40 cases were extracted from which effect sizes could be calculated (Table 1). These included spatial and temporal comparisons of raptor, corvid, gull, mustelid, fox and hedgehog predators and their effect on a range of game and non-game birds (waders, gulls and rails) and the brown hare (*Lepus europaeus*). Only two of the 40 cases concerned the brown hare, which resulted in their exclusion from two of the group analyses (Table 1).

To understand the impact of predator control on prey abundance, a meta-analysis was performed, using MetaWin Version 2 [30]. The effect size metric used was the log response ratio, defined as the ratio of the means measured in the experimental and the experimental control, i.e. prey abundance with and without predator removal (Appendix S3, Equation 1). A response ratio (ln R) and variance (*v*_i_) (Appendix S3, Equation 2) were calculated for each case in the meta-data set. The response ratio metric is biologically more relevant than other metrics e.g. hedges' *d*, that is most commonly used and which does not yield a clear biological interpretation [31]–[32].

The cases were analysed using a fixed effects model assuming no structure, to produce an overall mean effect size (Appendix S3, Equation 3). A 95% C.I. was calculated around this mean effect size, to identify any significant departure from no effect. A total heterogeneity statistic, which tests for heterogeneity in effect sizes between studies, was also produced (Appendix S3, Equation 4).

Our meta-data set included both temporal studies (*n* = the number of years) and spatial studies (*n* = number of sites) over which the mean abundance of prey species was calculated. The meta-analysis weighted temporal studies by the study length (from 3 to 10 years), as mean effect size is weighted by inverse variance (1/*v_i_*) which incorporates *n_E_* (experimental treatment years) and *n*_C_ (control treatment years) (Appendix S3, Equation 2). Spatial studies were likewise weighted by the number of sites; however, spatial studies also had a time component. To give added weight to longer studies and to standardise the data across spatial and temporal ones, we multiplied each spatial study effect size by the square root of its duration (1–3 years). This would make the response ratio (ln R) either more positive or more negative.

### Sub-group analyses

Biological and methodological factors were identified that might explain significant heterogeneity in effect sizes between cases (Table 1). Different predator taxa may impact prey abundance in different ways and were therefore described as *Bird* (one or two avian predator species controlled), *Mammal* (one mammal predator was controlled), or *Multiple* (a combination of predator species controlled). Likewise, different prey species may respond differently to predator removal, so the prey in each case were split into *Game Birds* (Galliformes), *Gulls and Terns* (Laridae), or W*ading Birds* (Scolopacida). There were insufficient cases of the remaining prey taxa, rails and mammals, to include them in this analysis (Table 1).

Prey status may also affect their response to predation, for instance declining species may respond differently from species at equilibrium. We therefore used the red and amber lists of [33] to classify prey species as *Red* (high conservation concern), *Amber* (medium conservation concern) and *No Designation* (not of conservation concern). Attempts were made to standardise the abundance measure to breeding pairs (of most interest for conservation), but this would have limited the number of studies included further. Therefore, abundance was classified according to the measure used: counts of *Individuals*, counts of *Pairs* (breeding pairs), and *Productivity* (no. chicks fledged, chicks per female), to see if this caused any variation in effect size. As the method of predator control is a further possible cause of heterogeneity in the data set, cases were split by those that controlled predators using Game keepers and those that did not.

Island species in the UK have been shown to be vulnerable to predation, especially from introduced species that can decimate breeding populations of ground nesting birds (e.g. [34]). We therefore classified each case as either *Island* or *Mainland*. We also identified two features of experimental design which could influence heterogeneity in effect sizes. Cases were as either *Spatial* (comparisons across sites with and without predators) or *Temporal* (comparisons over years differing in predator presence), and on the basis of the nature of predator removal, studies comparing systems before and after natural predator arrival (*BA*), as controlled experiments (*CE*), or as field studies with single predator removal (*FS*).

We calculated mean effect sizes for each level of each factor using random effects meta-analyses for categorical data (Appendix S3, Equation 5). Heterogeneity in effect sizes between factor levels was assessed using the test statistic Q_M_ (Appendix S3, Equation 6). Tests were complicated by low case representation in some factor levels, and especially due to non-independence of the factors being analysed (Table 1). We present results for representative factors for which the d.f. were large enough (3 or more) for meaningful analysis. As these analyses are exploratory, we discuss the results of tests uncorrected for multiple analyses and present 95% C.I.s for mean effect sizes. However, for information we also present adjusted significance levels calculated using sequential Bonferroni corrections (Figure 2, [35]).

Publication bias is a concern in literature reviews and can arise through the under-reporting of statistically insignificant results. Publication bias should not be a major problem here, as outcomes of, particularly long-term, manipulation experiments should be of interest whatever the result. We also incorporate results published in the grey literature, where most long-term monitoring data and small studies with non-significant results tend to be published [36]. However, there is likely to be bias in the species studied, as predators thought to cause a problem or of conservation concern will be targeted. Therefore, publication bias was tested by exploring the data graphically using a funnel plot [37] plotting the effect size for each case against its sample size. A funnel shaped plot (large opening at the smallest sample sizes) indicates that variation around the mean effect size decreases as sample size increases [30]. We also calculated a fail safe number [21] for the meta-data set using the fail-safe number calculator [38] to estimate the number of non-significant, unpublished and or missing studies that would be needed to change the significant result to a non-significant result.

## Supporting Information

Figure S1 A funnel plot with the large opening at the smallest sample sizes indicating that the variation around the mean effect size decreases as sample size increases. This suggests there is no bias in the reporting of results.
(1.85 MB TIF)

Appendix S1 (0.07 MB DOC)

Appendix S2 (0.03 MB DOC)

Appendix S3 (0.09 MB DOC)
