# Supplementary figures and images for: Meta-Analysis of the Effects of Predation on Animal Prey Abundance: Evidence from UK Vertebrates
Source: PLoS One. 2008 Jun 11;3(6):e2400. doi: 10.1371/journal.pone.0002400 (PMC2405933; doi:10.1371/journal.pone.0002400)

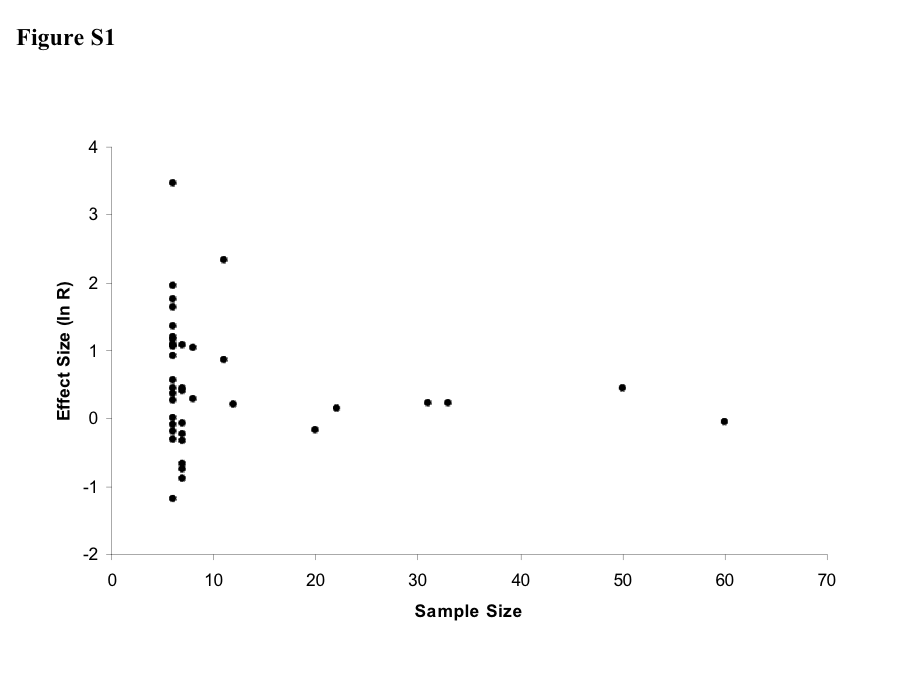

Supplement: Figure S1 — A funnel plot with the large opening at the smallest sample sizes indicating that the variation around the mean effect size decreases as sample size increases. This suggests there is no bias in the reporting of results. (1.85 MB TIF) [file pone.0002400.s001.tif]
