# Supplementary material for: Meta-Analysis of the Effects of Predation on Animal Prey Abundance: Evidence from UK Vertebrates
Source: PLoS One. 2008 Jun 11;3(6):e2400. doi: 10.1371/journal.pone.0002400 (PMC2405933; doi:10.1371/journal.pone.0002400)
Supplement: Appendix S1 — (0.07 MB DOC) [file pone.0002400.s002.doc]

**Appendix S1**

Table S1. Experiments and field studies of predator impact on prey that were reviewed but did not meet all the criteria for inclusion in the meta-analysis

| **Study** | **Predator** | **Type of experiment** | **Prey** | **Trend** |
| --- | --- | --- | --- | --- |
| [56] | Hooded crow | Predator removal | Hen Harrier | No effect of predator removal. |
| [57] | Gulls | Predator reduction | Puffins | Increase in prey recruitment with predator removal. |
| [58] | Weasels | Predator reduction | Field vole | Predator removal increased prey survival. |
| [59] | Gulls | Predator reduction | Oystercatchers | Increase in breeding pairs after cull commenced. |
| [60] | Hedgehogs | Predator reduction | Dunlin, lapwing, snipe and redshank | Nest success of prey higher when predator absent. |
| [61] | Cormorants | Unreplicated predator control | Fish | Only small amount of fish loss attributable to cormorants. |
| [62-63], [2] | Hen harriers | Different levels of predation | Red grouse | Predation limited the grouse population and suppressed a cycle. |
| [64]  **mid-term results** | Multiple | Predator removal | Passerines | Decrease in prey abundance with predator removal. |
| [65] | Multiple | Predator removal | Grey partridge | Decrease in prey densities with predator removal. |

**References**

56 Amar A, Redpath SM (2002) Determining the cause of the hen harrier decline on the Orkney Islands: an experimental test of two hypotheses. Anim Conserv 5: 21-28.

57 Finney SK, Harris MP, Keller LF, Elston DA, Monaghan P et al. (2003) Reducing the density of breeding gulls influences the pattern of recruitment of immature Atlantic puffins *Fratercula arctica* to a breeding colony. J Appl Ecol 40: 545-552.

58 Graham IM, Lambin X (2002) The impact of weasel predation on cyclic field-vo8e survival: the specialist predator hypothesis contradicted. J Anim Ecol 71: 946-956.

59 Harris MP, Wanless S (1997) The effect of removing large numbers of gulls *Larus* spp. On an island population of oystercatchers *Haematopus ostralegus*: implications for management. Biol Conserv 82: 167-171.

60 Jackson DB (2001) Experimental removal of introduced hedgehogs improves wader nest success in the Western Isles, Scotland. J Appl Ecol 38: 802-812.

61 Pilcher MW, Feltham MJ (1997) An assessment of cormorant predation on stillwater coarse fish populations in the Lea and Colne valleys of the Thames catchment. Environment Agency (Thames NE area) R&D Technical Report W101.

62 Redpath SM, Thirgood SJ (1997) Birds of prey and grouse. The Stationary Office, London.

63 Thirgood SJ, Redpath SM, Rothery P, Aebischer, NJ (2000) Raptor predation and population limitation in red grouse. J Anim Ecol 69: 504-516.

64 Stoate C, Szczur J (2005) Predator control as part of a land management system: impacts on breeding success and abundance of passerines. Wildl Biol Prac 1: 53-59.

65 Tapper SC, Green RE, Rands MRW (1982) Effects of mammalian predators on partridge populations. Mammal Rev 12: 159-167.
