# Supplementary material for: Meta-Analysis of the Effects of Predation on Animal Prey Abundance: Evidence from UK Vertebrates
Source: PLoS One. 2008 Jun 11;3(6):e2400. doi: 10.1371/journal.pone.0002400 (PMC2405933; doi:10.1371/journal.pone.0002400)
Supplement: Appendix S2 — (0.03 MB DOC) [file pone.0002400.s003.doc]

**Appendix S2**

**Search strategy**

The following electronic databases were searched: BIOSUS via EDINA, **BOPCAS: British Official Publications Current Awareness Service,** Copac, Index to Theses Online (1970-2005), ISI Web of Knowledge (comprising of ISI Web of Science: Science Citation Index Expanded 1945-present and ISI Proceedings: Science and Technology Proceedings 1990-present), JSTOR, Scirus, **Sigle via ARC2 WebSPIRS,** English Nature’s ‘WildLink’ and Countryside Council of Wales (CCW) library (providing access to grey literature of relevance to EN and CCW respectively).

Publication searches were also conducted using the internet meta-search Google Scholar and Dogpile with the first 100 word documents or PDF hits from each website were examined for appropriate literature or data. In addition, the following statutory and non-governmental organisation websites were inspected: UK Department for Environment, Farming and Rural Affairs (Defra) and Scottish Natural Heritage (SNH). The libraries of RSPB, Natural England, Central Science Laboratory and Game Conservancy Trust were hand searched for further appropriate literature.

**Review process**

Studies collected for each of the questions underwent a three-fold filter process before being accepted for meta-analysis. Initially, all articles from the search were filtered by title and any obviously irrelevant studies were rejected. **Two independent reviewers assessed the title and abstract of a random subset of studies for inclusion in the review on a consensus basis. The degree of agreement between the two reviewers on this subset (approximately 25% of the studies) was tested statistically.** The most desirable way of understanding the impact of predation on prey abundance is through controlled predator removal experiments where prey abundance from the experimental and control areas are compared, where it is possible to be confident that any resultant change in abundance is due to predation. Therefore, s**tudies were accepted for full text viewing if they appeared from the title and abstract to be replicated controlled experiments. However, studies were also considered appropriate if they were well conducted field studies which compared sites or years where a predator was present and absent and had measured the abundance of prey species using either density, individual counts, breeding pairs, or productivity,** or if the abstract was ambiguous and did not allow inferences to be drawn about the content of the article. Finally, the full text of all remaining studies was checked by the two reviewers and, on the same criteria, either rejected or accepted for meta-analysis.
