# Supplementary material for: Meta-Analysis of the Effects of Predation on Animal Prey Abundance: Evidence from UK Vertebrates
Source: PLoS One. 2008 Jun 11;3(6):e2400. doi: 10.1371/journal.pone.0002400 (PMC2405933; doi:10.1371/journal.pone.0002400)
Supplement: Appendix S3 — (0.09 MB DOC) [file pone.0002400.s004.doc]

**Appendix S3**

**Meta-analyses models**

The following equations were derived from [30].

ln *R =* ln Equation 1.

Where $\bar{X}^{E}$ is the mean prey abundance of the experimental (predator removal or absence) treatment and $\bar{X}^{C}$is the mean of the control treatment.

*v*_i_ = + Equation 2.

Where *sd* and *n* are the standard deviation and the sample size of the experimental and control means.

Using a fixed effects model the cumulative (or weighted average) response ratio is calculated as:

Equation 3.1

where the weight for the *i*^th^ study is the reciprocal of its sampling variance – *w*_i_ = 1/*v*_i_, *n*  is the number of studies and ln R_i_ is the effect size for the *i*^th^ study.

The variance of is a function of the individual weights and is calculated as:

Equation 3.2

The confidence interval is calculated using:

CI = ± Equation 3.3

Where *t* is the two-tailed critical value from the Student’s *t*-distribution at the critical level $\alpha$.

The total heterogeneity of the data is:

Q_T_ = Equation 4.

where *n* is the number of studies in the analysis and In R_i_ is the response ratio of the i^th^ estimate and each study is weighted by inverse variance (*w*_i_ = 1/*v*_i_).

For the random effects model (for categorical data) the average response ratio was calculated for each group within each factor () and was calculated as:

= Equation 5.1

where *k_j_* is the number of studies in the *j*^th^ group.

The variance of is:

Equation 5.2

and the confidence interval around is calculated as:

CI = Equation 5.3

The between group heterogeneity (difference among group cumulative response ration) is calculated using:

Q_M_ = Equation 6.

where *m* is the number of groups in the factor being analysed, ln R_j_ is the response ratio for the *j*^th^ group and is the cumulative response ratio. The significance level of Q_M_ is determined by testing against a χ^2^ distribution with 1 degree of freedom.

Note - Q_M_ may be referred to as Q_B_ in other meta-analyses.
